# Synthesis of Porous Hierarchical In_2_O_3_ Nanostructures with High Methane Sensing Property at Low Working Temperature

**DOI:** 10.3390/nano12173081

**Published:** 2022-09-05

**Authors:** Huiju Zhang, Jiangnan Chang, Yan Wang, Jianliang Cao

**Affiliations:** 1 College of Chemistry and Chemical Engineering, Henan Polytechnic University, Jiaozuo 454000, China; 2 State Collaborative Innovation Center of Coal Work Safety and Clean-Efficiency Utilization, Henan Polytechnic University, Jiaozuo 454000, China

**Keywords:** porous hierarchical In_2_O_3_ nanostructure, gas sensor, methane, low temperature, gas sensing performance

## Abstract

Different hierarchical porous In_2_O_3_ nanostructures were synthesized by regulating the hydrothermal time and combining it with a self-pore-forming method. The gas-sensing test results show that the response of the sensor based on In_2_O_3_ obtained after hydrothermal reaction for 48 h is about 10.4 to 500 ppm methane. Meanwhile, it possesses good reproducibility, stability, selectivity and moisture resistance as well as a good exponential linear relationship between the response to methane and its concentration. In particular, the sensor based on In_2_O_3_ can detect a wide range of methane (10~2000 ppm) at near-room temperature (30 °C). The excellent methane sensitivity of the In_2_O_3_ sensor is mainly due to its unique nanostructure, which has the advantages of both porous and hierarchical structures. Combined with the DFT calculation, it is considered that the sensitive mechanism is mainly controlled by the surface adsorbed oxygen model. This work provides a feasible strategy for enhancing the gas sensitivity of In_2_O_3_ toward methane at low temperatures.

## 1. Introduction

Methane (CH_4_) is a colorless, odorless, flammable, explosive, and suffocating gas. When the concentration is high, methane will cause headache, nausea, vomiting and other adverse phenomena [1,2,3,4,5]. In coal mines, methane is the main component that causes gas outburst and explosion. When the content of methane in air reaches 5–15%, gas explosion will occur if there are ignition factors [1,2,3]. In order to ensure the safety of coal mine production, the content of methane in the coal mine gas must be monitored in real time and accurately. At present, the monitoring index of methane content in coal mine gas is 0.5% (5000 ppm). That is to say, when the methane content reaches 0.5%, an alarm will be given to ensure the safe evacuation of field personnel. Further, the main component of domestic natural gas is methane. Once the natural gas leaks, it will pose a serious threat to the safety of people’s lives and property. In the field of the coal chemistry industry, the leakage of natural gas in factories will cause serious pollution in the atmosphere. Thus, real-time monitoring of methane is very important. Simultaneously, methane is a non-polar gas molecule with a stable symmetrical structure and inert, so it is difficult to monitor methane effectively in real time. Up to now, the commonly used methane sensors have some common limitations [1,6]: high working temperature, intrinsic safety problems, high energy consumption and poor stability. Therefore, it is necessary to develop methane sensors with the characteristics of high sensitivity, low working temperature, low energy consumption and high stability.

Metal oxide semiconductors (MOSs) have attracted extensive attention and vigorous development due to their good chemical and thermal stability along with excellent electrical and optical properties. MOSs are widely used in energy storage, sensor technology, sterilization and purification, catalysis, chemical industry, etc. [7,8]. According to the different types of carriers, MOSs are generally divided into an *n*-type semiconductor and *p*-type semiconductor. Among them, *n*-type semiconductors include ZnO, SnO_2_, In_2_O_3_, and *p*-type semiconductors include Co_3_O_4_, NiO, Cr_2_O_3_, etc. Due to the unique properties of semiconductor carriers, they are widely used in the fields of electricity, photochemistry, magnetism, catalysis and so on. Among them, In_2_O_3_ has a band gap of 3.6 eV [9]. It is the main material for manufacturing Indium Tin Oxides (ITO) and is widely used in liquid crystal displays and other fields. At the same time, In_2_O_3_ has good gas sensing performance and is also widely used in the gas sensor field [10].

At present, research on improving the gas sensing performance of sensors is mainly carried out from the aspects of morphology control and doping modification of sensitive materials. It has been reported that the specific structures contribute to the improvement in gas sensing properties, and the mechanisms of different structures are different. The factors affecting the gas sensing properties of MOSs are not only energy levels, but also carrier conduction and gas sensing active sites. Especially, porous materials are widely studied because of their excellent properties, including porosity, high specific surface area and a large number of active sites. Generally, the main methods of synthesizing porous materials are the soft template method [11], hard template method [12] and self-pore-forming method [13]. In addition to the classical template method, the self-pore-forming method has gradually attracted the attention of researchers. The self-pore-forming method requires the synthesis of metal–organic covalent compounds, metal alkoxides, carbonates and other materials. These self-pore-forming materials are usually referred to as “precursors”. During sintering in air, unstable components in the precursor will decompose, and the remaining components will further form stable oxides. This process produces a large amount of gas, leading to the formation of porous structures. This method can generate a pore structure in situ and crystallize at the same time as the solid-phase transformation reaction of raw materials. Since there is not enough concentration of raw materials around the grains for growth, crystal particles are generally small and there are many surface defects, which can effectively improve the gas sensitivity of the as-prepared samples.

In this study, InCl_3_·4H_2_O was used as In source to synthesize the In(OH)_3_ precursor by hydrothermal method, and then porous hierarchical In_2_O_3_ nanostructures were prepared by the self-pore-forming method. The evolution and formation of the morphology of In_2_O_3_ were studied by controlling the hydrothermal reaction time. In addition, we tested the gas-sensing performances of the In_2_O_3_ sensor to CH_4_ at low temperature. Meanwhile, we used the DFT method to study the adsorption characteristics of In_2_O_3_ to CH_4_ and analyzed the relationship between surface adsorption oxygen and gas-sensing performance.

## 2. Materials and Methods

### 2.1. Chemicals

Indium (III) chloride tetrahydrate (InCl_3_·4H_2_O, 99.0%) was purchased from Shanghai Civic Chemical Technology Co., Ltd., Shanghai, China. Citric acid (C_6_H_8_O_7_, 99.5%) and urea (CO(NH_2_)_2_, 99.5%) was purchased from Tianjin Kermel Chemical Reagent Co., Ltd., Tianjin, China. Ethylene glycol (EG, 98%) and ethanol (EtOH, 99.5%) were purchased from Shanghai Macklin Biochemical Co., Ltd., Shanghai, China. All reagents are analytical grade and used without further purification.

### 2.2. Material Synthesis

Porous In_2_O_3_ nanostructures with regular morphologies were synthesized via the one-step hydrothermal combined with later self-pore-forming method by controlling hydrothermal reaction time. Firstly, 1 mmol of InCl_3_·4H_2_O was dissolved in 60 mL of mixed solution (EG and H_2_O 30 mL each) under continuous stirring. Then, 2.5 mmol of C_6_H_8_O_7_ and 15 mmol of CO(NH_2_)_2_ were added to the above mixed solution, respectively. After continuous stirring for 1 h, the obtained homogeneous solution was sealed in 50 mL of a Teflon-lined stainless-steel autoclave and hydrothermal treatment at 160 °C for different times (18 h, 24 h, 48 h). After the reactor was naturally cooled to room temperature, the precursor products were collected by centrifugation and washed several times with H_2_O and EtOH. Finally, porous In_2_O_3_ nanostructures with regular morphology were obtained by self-pore-forming calcination in air at 400 °C for 4 h. According to the order of hydrothermal reaction time from short to long, the final samples were named 18-In_2_O_3_, 24-In_2_O_3_ and 48-In_2_O_3_, respectively.

### 2.3. Material Characterization

Powder X-ray diffraction was carried out on a Bruker/D8-Advance diffractometer (Bruker, Ettingen, Germany) (Cu Kα radiation, λ = 0.15418 nm), the scanning range of data collection was 10–90° (2*θ*) and was in the continuous mode with step size of 0.02° (2*θ*). The morphology and structure were characterized by field emission electron microscopy (FESEM, FEI Quanta 250 FEG, Hillsboro, OR, USA) and transmission electron microscopy (TEM, JEOL, JEM-2100, Akishima, Tokyo, Japan). The Brunauer–Emmett–Teller (BET) specific surface area testing was performed on a Quantachrome Autosorb-iQ sorption analyzer (Quantachrome, Boynton Beach, FL, USA), and the pore size distribution was estimated by the Discrete Fourier Transform (DFT) method. X-ray photoelectron spectroscopy (XPS) characterization was carried out by Al-Kα radiation through Thermo Scientific K-Alpha+ XPS (Thermo Fisher Scientific, Waltham, MA, USA), the shift in the binding energy owing to the relative charge was corrected using the C 1 s peak at 284.8 eV as an internal standard. The thermal analysis was carried out on a thermogravimetric analyzer of Setaram evolution 2350 (Setaram, Lyon, France), heated from 30 °C to 600 °C in an air stream at a heating rate of 10°·min^−1^.

### 2.4. Sensor Fabrication and Measurement

The details of sensor fabrication are similar to our previous reports [14,15]. Firstly, the three obtained samples of 18-In_2_O_3_, 24-In_2_O_3_ and 48-In_2_O_3_ were ground into fine powder. Then, the samples were separately prepared into dispersions (1 mg in 2 mL H_2_O) and drop-coated on three sensor substrates with Ag-Pd interdigital electrodes. The sensor prepared before the gas sensing test was stable for 12 h at 60 °C. The sensitivity of 18-In_2_O_3_, 24-In_2_O_3_ and 48-In_2_O_3_ sensors (R_a_/R_g_) was tested by an intelligent gas-sensing analysis system of CGS-4TPS (Beijing Elite Tech Co., Ltd., Beijing, China) under laboratory conditions (20% RH, 28 °C). Typically, a certain volume of CH_4_ is injected into a closed chamber with a syringe. The CH_4_ gas is fully in contact with the electrode under the action of an inset fan. When the resistance reaches a stable value, the chamber is opened and the electrode is re-exposed to air. At this time, when the resistance value remains stable again in the air, the signal is collected. 

## 3. Results and Discussion

### 3.1. Sample Characterization

**Figure 1:**
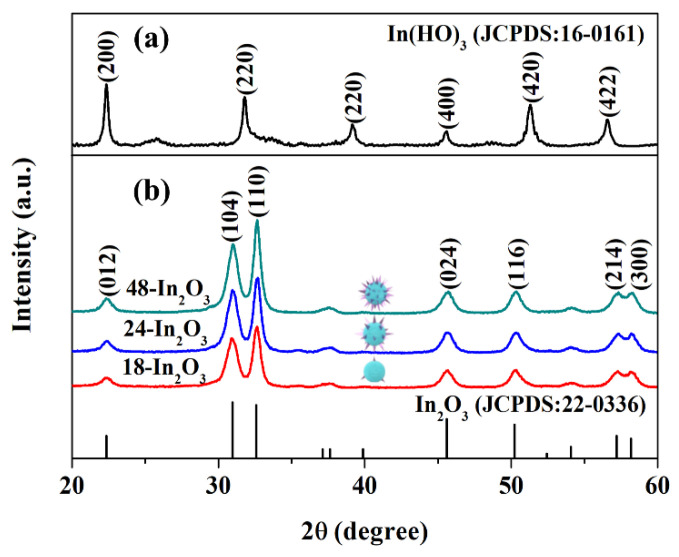
XRD pattern of (**a**) In(OH)_3_ precursor and (**b**) porous In_2_O_3_ samples.

The purity and phase structure of the samples were characterized by XRD. As shown in Figure 1a, the peaks located at 22.29°, 32.28°, 45.42°, 51.82° and 55.82° are assigned to the (200), (220), (400), (420) and (422) crystal facets of In(OH)_3_. All diffraction peaks are consistent with the cubic phase In(OH)_3_ (JCPDS No. 16-0161), revealing that the first step of hydrothermal reaction formed the In(OH)_3_ phase. As In(OH)_3_ can be decomposed into In_2_O_3_ at high temperature, In(OH)_3_ obtained by the hydrothermal method is used as the precursor for the formation of In_2_O_3_. According to the TG-DTA analysis result (Figure S1), 400 °C is selected as the self-pore-forming calcination temperature to synthesize In_2_O_3_. One can see from Figure 1b that 18-In_2_O_3_, 24-In_2_O_3_ and 48-In_2_O_3_ all have diffraction peaks at 22.37, 30.99, 32.61, 45.61, 50.25, 57.2 and 58.19°, which can be well indexed to the (012), (104), (110), (024), (116), (214) and (300) crystal facets of hexagonal structure of In_2_O_3_ (JCPDS No. 22-0336), respectively. No other diffraction peaks were found in the XRD patterns, which indicated that the prepared samples possess high purity. At the same time, we observed that the XRD peak intensity of the samples increases gradually with the prolongation of hydrothermal reaction time, which not only improves the crystallinity of the samples, but also affects the average crystallite size and lattice strains of the samples. The average crystallite size (D) and lattice strains (ε) of three samples were calculated by Debye–Scheller Formulas (1) and (2).

D = Kλ/(β cos *θ*) (1)

ε = (β cos *θ*)/4 (2)

where K (K = 0.89) is the Scherrer constant, D is the crystallite size, λ (λ = 0.15418 nm) is the X-ray wavelength, β is the full width half maximum (FWHM) of the (110) peak and *θ* is the Bragg diffraction angle. The calculation results are listed in Table 1. It is evident from the data in Table 1 that, with the prolongation of the hydrothermal reaction time, the crystallite size gradually increases and the lattice strains gradually decrease. These conclusions also verify the phenomena observed from the XRD pattern.

**Figure 2:**
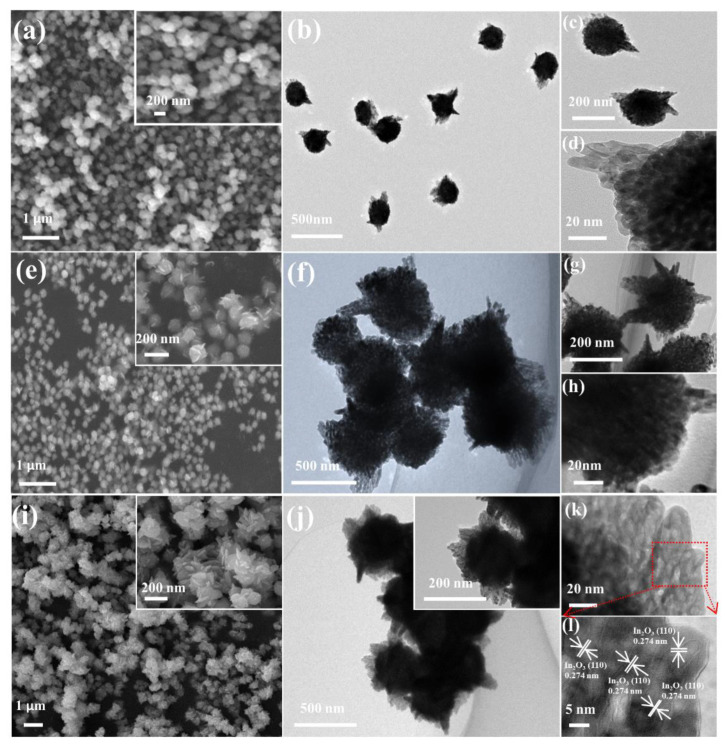
(**a**) FESEM image and (**b**–**d**) TEM image of the 18-In_2_O_3_; (**e**) FESEM image and (**f**–**h**) TEM image of the 24-In_2_O_3_; (**i**) FESEM image, (**j**,**k**) TEM image and (**l**) HRTEM image of the 48-In_2_O_3_.

The morphology and nanostructure of porous In_2_O_3_ were characterized by FESEM and TEM. The FESEM image of 18-In_2_O_3_ (Figure 2a) shows that the sample is composed of innumerable monodisperse nanospheres. Further characterization by TEM reveals that the morphology of 18-In_2_O_3_ is similar to that of tadpoles, with a tail on each spherical particle (Figure 2b,c). The porous structure of the nanospheres can be inferred from the contrast of light and dark in Figure 2d. The FESEM image of 24-In_2_O_3_ is shown in Figure 2e. At low magnification, they are still like monodisperse nanospheres. At high magnification, many teeth grow on the surface of nanospheres. TEM characterization clearly shows that 24-In_2_O_3_ is composed of spherical structures such as gears (Figure 2f,g). The FESEM image of the 48-In_2_O_3_ sample (Figure 2i) shows that it is no longer like monodisperse nanospheres. A further observation result by TEM shows that the nanospheres are surrounded by full wings and cross-linked, which looks like sunflowers in full bloom (Figure 2j). From Figure 2h,k, a light–dark contrast phenomenon similar to that of the 18-In_2_O_3_ (Figure 2d) is observed, which indicates that both 24-In_2_O_3_ and 48-In_2_O_3_ are porous materials. The HRTEM characterization and analysis results of 48-In_2_O_3_ are shown in Figure 2l. The lattice fringe of d = 0.274 nm corresponds to the (110) crystal face of hexagonal phase In_2_O_3_.

**Figure 3:**
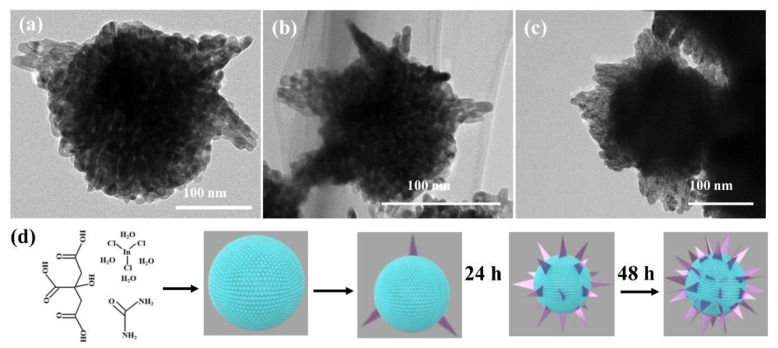
(**a**–**c**) FESEM images of the samples at various reaction times: (**a**) 18 h, (**b**) 24 h and (**c**) 48 h; (**d**) schematic of the growth process of the porous In_2_O_3_ nanostructures at various reaction times.

The evolution formation mechanism of porous In_2_O_3_ nanostructures has been investigated. The TEM images in Figure 3a–c are 18-In_2_O_3_, 24-In_2_O_3_ and 48-In_2_O_3_ samples obtained at different hydrothermal treatment times, respectively, which reveal the evolution of the morphology and nanostructure with different hydrothermal treatment times. During the initial 18 h hydrothermal treatment, porous nanospheres with rough and regular surfaces were formed, and then very few nanosheets were grown on the surface of In_2_O_3_ porous spheres (Figure 3a). With the prolongation of hydrothermal reaction time, the size and number of nanosheets increase gradually. When the hydrothermal reaction time reached 48 h, the sample exhibits a blooming sunflower morphology (Figure 3b,c). Based on the above observations of morphology and nanostructure changes, the possible evolution formation process of porous In_2_O_3_ spherical nanostructures with the extension of hydrothermal reaction time is presented in Figure 3d. Firstly, InCl_3_·4H_2_O was hydrolyzed to form In(OH)_3_ nanoparticles. Then, these nanoparticles aggregated into a spherical structure. After self-pore-forming calcination, In(OH)_3_ lost OH- forming In_2_O_3_, which makes the NSs have a porous structure. With the increase in hydrothermal treatment time, In(OH)_3_ nanocrystals aggregated into nanosheets grow further, and nanosheets grow gradually in order to minimize surface energy [16,17]. Moreover, it can be found that the size of microspheres (100~200 nm) assembled with small nanoparticles inside the In_2_O_3_ nanostructures gradually decreases with the increase in hydrothermal reaction time. However, the number of shell nanosheets of the In_2_O_3_ nanostructure gradually increases. The grain size of 18-In_2_O_3_, 24-In_2_O_3_ and 48-In_2_O_3_ is 14.69 nm, 14.78 nm and 15.65 nm, respectively, which is consistent with the SEM analysis result. The longer the hydrothermal treatment time, the more nanosheets grow. After calcination, porous hierarchical nanostructures were formed.

**Figure 4:**
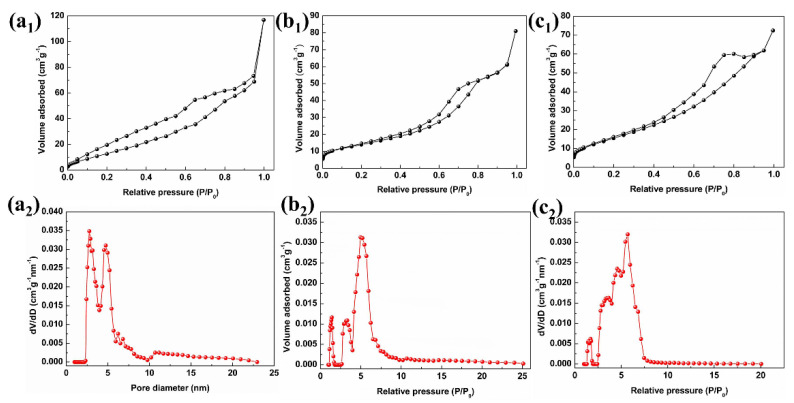
Typical N_2_ adsorption/desorption isotherms and pore size distribution curves of (**a**) 18-In_2_O_3_, (**b**) 24-In_2_O_3_ and (**c**) hierarchical 48-In_2_O_3_.

The specific surface areas of 18-In_2_O_3_, 24-In_2_O_3_ and 48-In_2_O_3_ samples were estimated through the date of N_2_ adsorption–desorption isotherms, as shown in Figure 4. For these three samples, the existence of hysteresis loops can be clearly observed from the nitrogen adsorption–desorption curves, which proves that the hierarchical nanomaterials possess mesoporous structure. From the pore size distribution curves corresponding to the three structures (insets in Figure 4), the distribution center shifts with the increase in hydrothermal reaction time. The specific surface areas of 18-In_2_O_3_, 24-In_2_O_4_ and 48-In_2_O_3_ are 47.258, 50.752 and 59.635 m^2^·g^−^^1^, respectively. Furthermore, according to the specific surface area characterization results, it is found that the specific surface areas and total pore volumes (0.087, 0.090 and 0.091 cm^3^·g^−^^1^) of the samples increase in turn with the prolongation of hydrothermal reaction time. Among the three samples, the 48-In_2_O_3_ has the largest specific surface area and total pore volume, which can provide more active sites for the adsorption and reaction of gas molecules and could further provide more effective methods for the internal transport of gas molecules and electrons. The porous structures of the as-prepared samples are essential for improving the gas sensitivity of the sensor.

### 3.2. Gas Sensing Properties Evaluation

**Figure 5:**
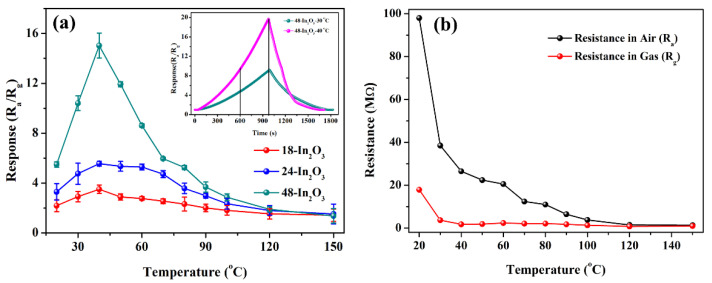
(**a**) Response of the porous In_2_O_3_ samples relative to operating temperature to 500 ppm of CH_4_ (the inset shows the response–recovery curve of 48-In_2_O_3_ to 200 ppm methane at 30 and 40 °C, respectively); (**b**) The change in resistance (R_a_ and R_g_) of the 48-In_2_O_3_ sensor in air and gas with the sensor working temperature.

It is well known that the working temperature is one of the important factors for affecting the gas sensing performance of the sensor. In order to determine the optimal working temperature, the functional relationship between the responses of sensors based on the samples of 18-In_2_O_3_, 24-In_2_O_3_ and 48-In_2_O_3_ to 500 ppm methane and the working temperature were tested, and the results are shown in Figure 5a. Within the range of 20–150 °C, the responses of these 3 sensors ranked as 18-In_2_O_3_ < 24-In_2_O_3_ < 48-In_2_O_3_, and the response curves of these 3 sensors exhibited a mountain-like change trend, and reached their maximum responses values at 40 °C. At low temperature, CH_4_ is relatively stable and does not easily cause potential safety hazards such as combustion and explosion. Therefore, methane sensors are bound to develop in the direction of low temperature, or even room temperature detection, and the sensitivity should also be considered. Taking 48-In_2_O_3_ sample as an example, when the temperature is changed around 40 °C, its response value changes significantly. The response values of the 48-In_2_O_3_ sensor at 30 °C, 40 °C and 50 °C are about 10, 16 and 12, respectively, which demonstrates that working temperature has a huge effect on the response of the sensors. This may be because the working temperature plays an important role in the dynamic equilibrium of adsorption, reaction and desorption of target gas molecules. If the temperature is too low, the activation energy is too low for CH_4_ molecules to fully react with the adsorption oxygen. As the temperature increases, CH_4_ molecules desorb on the surface of the material before the full reaction. From the Figure 5a, we can see that the response of the 48-In_2_O_3_ sensor to 500 ppm CH_4_ at 30 °C is still about 10. The inset in Figure 5a shows the real-time response–recovery curves of the 48-In_2_O_3_ sensor to 200 ppm methane at 30 and 40 °C. It can be seen that the extended response time of the sensor still cannot reach a stable platform, and the two curves have the same trend of change and the response–recovery speed is close. Therefore, in order to test whether the In_2_O_3_ sensor has good gas sensitivity to methane at near room temperature, 30 °C was chosen as the best working temperature to detect the next gas sensitivity, and the response time was uniformly controlled at 600 s. Figure 5b shows the variation in the resistance (R_a_ and R_g_) of the 48-In_2_O_3_ sensor with the working temperature. With the working temperature increases, the value of R_a_ decreases continuously, which may be related to the semiconductor property of In_2_O_3_ [18,19]. When the 48-In_2_O_3_ sensor is exposed to 500 ppm methane gas, the R_g_ value is also reduced, which may be due to the reducing nature of methane gas [19]. The largest change in the resistance of the sensor is from R_a_ = 26.5 MΩ to R_g_ = 1.763 MΩ at 40 °C. The large change in the resistance of the sensor (R_a_ to R_g_) results in an enhanced response of the 48-In_2_O_3_ sensor to methane.

**Figure 6:**
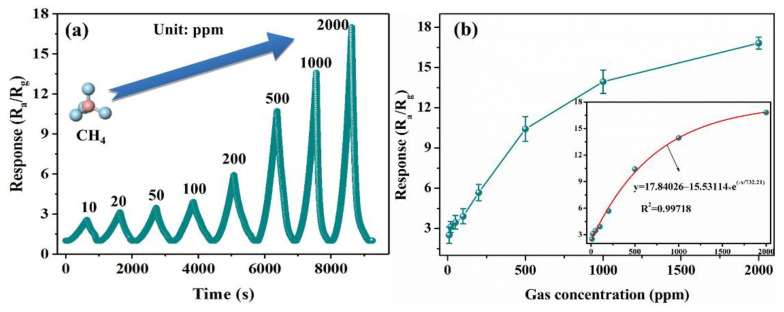
(**a**) Dynamic response–recovery for the 48-In_2_O_3_ sensor toward CH_4_ in a concentration range of 10–2000 ppm at 30 °C; (**b**) the response of the sensor to the concentration of CH_4_ in the range of 10–2000 ppm (the bottom right corner inset is an exponential relationship between the gas response of the sensor and the gas concentration).

The dynamic response–recovery curve (Figure 6a) shows that the response of the 48-In_2_O_3_ sensor increases with the increase in methane concentration from 10 to 2000 ppm, and it has better response–recovery characteristics. The sensor works well in the whole range of CH_4_ concentration. In order to better judge the relationship between sensor response and CH_4_ concentration, sensor response versus methane concentration was studied in Figure 6b. The response increases rapidly with the increase in CH_4_ concentration until 500 ppm, indicating that the sensor can detect CH_4_ in a wider concentration range. When CH_4_ concentration is more than 500 ppm, the increasing trend in response slows down, which may be due to the gradual saturation of the CH_4_ molecule on the sensor surface. The relationship between CH_4_ concentration and the response values can be expressed by the following expression: *y* = 17.84026 − 15.53114e^(−*x*/732.21)^, where y represents the response and *x* represents the concentration of CH_4_. The fitting curve exhibits a good exponential relationship (*R*^2^ = 0.99718), which facilitates the practical application of the sensor. The responses of the sensor based on 48-In_2_O_3_ to 10, 20, 50, 100, 200, 500, 1000 and 2000 ppm CH_4_ are 2.5, 3, 3.5, 4, 6, 10.5, 13.5 and 16.7, respectively. 

**Figure 7:**
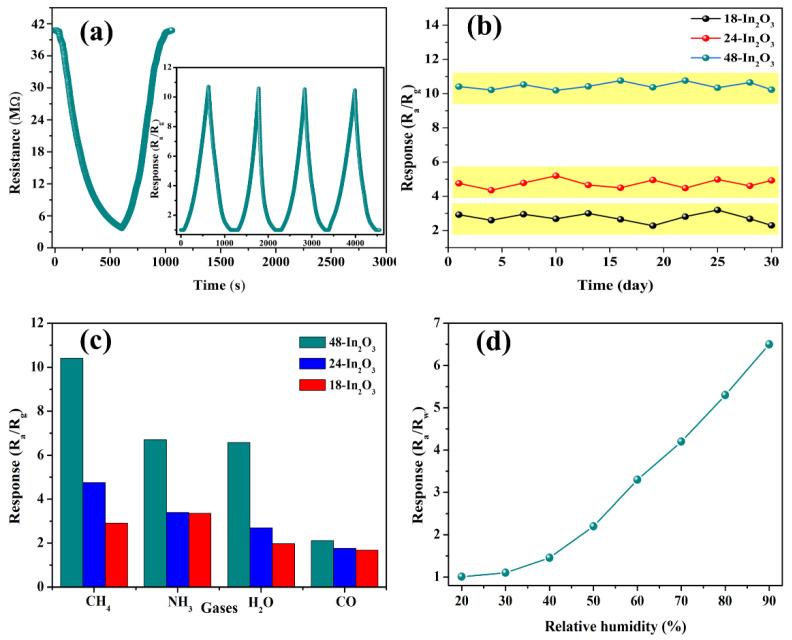
Response of the 48-In_2_O_3_ sensor (**a**) to 500 ppm CH_4_ (the inset is cyclic measurements); (**b**) tested days to 500 ppm CH_4_; (**c**) response of In_2_O_3_ sensors to 500 ppm of CH_4_, CO, NH_3_ H_2_O and 90% RH air; (**d**) response of the 48-In_2_O_3_ sensor to different RH air at 30 °C.

Figure 7a shows the dynamic response characteristics of the resistance of the 48-In_2_O_3_ sensor to 500 ppm CH_4_. It can be seen that after CH_4_ gas injection, the resistance of the sensor begins to decrease, and the corresponding response value increases; when the resistance reaches the lowest value, the corresponding response value decreases, which is consistent with the characteristics of the transient response recovery curve in Figure 6a. The repeatability of the 48-In_2_O_3_ sensor was studied by four cycles of dynamic response–recovery test (inset in Figure 7a), the initial value of the sensor remains basically unchanged, and the response to 500 ppm CH_4_ is basically consistent, indicating that the sensor has good repeatability. The long-term stability of the sensor was evaluated by recording the response of sensors to 500 ppm CH_4_ for 30 days. As shown in Figure 7b, the sensors based on 18-In_2_O_3_, 24-In_2_O_3_ and 48-In_2_O_3_ all remain relatively stable response values with little fluctuation during the 30-day test. In particular, the response value of the 48-In_2_O_3_ sensor is between 10 and 11. In addition, the relative standard deviation is less than 7%, which demonstrates that the sensor based on 48-In_2_O_3_ has a good long-term stability. The selectivity of the 18-In_2_O_3_, 24-In_2_O_3_ and 48-In_2_O_3_ sensors to 500 ppm CH_4_, ammonia solution (NH_3_ concentration is 25–28%), carbon monoxide (CO/100%) and 90% relative humidity (RH) of air was also investigated (Figure 7c). We chose these gases for interference testing for the following reasons: (1) NH_3_ is also one of the most toxic and harmful gases in coal mines, which can seriously damage the skin and upper respiratory tract of the miners; (2) Under coal mines, coal’s spontaneous combustion releases a large amount of CO, which is one of the main factors causing coal mine fire hazards; (3) Mine air is relatively humid and has a high relative humidity (RH%). Therefore, we need to test the interference of moisture on CH_4_ selectivity. As a result, the 48-In_2_O_3_ sensor has a higher response to all gases than the other two sensors and has the highest response to CH_4_, indicating that the 48-In_2_O_3_ sensor has relatively good selectivity to CH_4_. In addition, for 48-In_2_O_3_, it can be concluded that the coefficient of selectivity (K = S_CH4_/S_interference_ gases) of CH_4_ to NH_3_, H_2_O and CO are 1.54, 1.61 and 4.2, respectively, which demonstrates that the sensor based on 48-In_2_O_3_ has good selectivity. Selectivity is the most important indicator to evaluate whether the sensor can be put into practical application. Therefore, improving the selectivity of sensors is the focus of many researchers. There are several ways to improve the selectivity of sensors, such as temperature modulation and the multisensory arrays. In follow-up research, we will also try to adopt these strategies to further improve the selectivity of our sensors.

In order to further study the influence of humidity on the sensor, the response (R_a_/R_w_) of the sensor under different relative humidity (RH) air at 30 °C was detected, as shown in Figure 7d. Obviously, when RH < 30%, the response of the sensor is very small or even can be ignored. The responses of the sensor based on 48-In_2_O_3_ to CH_4_ were 1.0, 1.2, 1.5, 2.2, 3.5, 4.3, 5.5 and 6.5 at 20 RH%, 30RH%, 40RH%, 50RH%, 60RH%, 70RH%, 80RH% and 90RH%, respectively. The responses increase with the increase in the RH%. In general, the responses of most metal oxide gas sensors decrease with the increasing relative humidity. This is because water molecules react with adsorbed oxygen to generate inactive hydroxyl groups, which consumes a portion of active sites on the surface of the sensing materials. However, our result was opposite. This is probably related to the mesophase produced in the redox reaction of CH_4_ gas. Later, we can use some in situ characterizations to verify the role of water molecules in the CH_4_ oxidation process. It shows that the 48-In_2_O_3_ sensor has relatively good moisture resistance, but it is still a certain challenge to apply it to the ultra-high humidity environment, and we need more in-depth and comprehensive research.

Taken together, the above results show that 48-In_2_O_3_ not only has a low working temperature, but also has high sensitivity, stability, selectivity and humidity resistance. Moreover, the 48-In_2_O_3_ CH_4_ sensor is more competitive in response and working temperature than most reported CH_4_ sensors (Table 2) [18,19,20,21,22,23,24,25,26]. The results reveal that the 48-In_2_O_3_ CH_4_ sensor has better practical application potential.

### 3.3. Gas Sensing Mechanism

At present, the surface adsorption oxygen model is still mainly used to explain the sensitive mechanism of metal oxide semiconductors [15,18,19,20,27,28]. The resistance change caused by the reaction between the target gas molecules and the chemisorbed oxygen determines the response value [29,30,31]. In_2_O_3_ is a typical *n*-type semiconductor. When the In_2_O_3_ sensor is in the air, the oxygen molecules in the air will be adsorbed on the surface of the sensitive material. With the rise in temperature, the oxygen molecules will capture the electrons in the conduction band of the material to form oxygen negative ions, such as Formulas (3) and (4), which will increase the resistance of the In_2_O_3_ sensor in the air. When the methane gas molecules are brought into contact with the surface of the In_2_O_3_ sensitive material, it will react with oxygen anion and release the captured electrons back to the conduction band of the material, such as Formulas (5) and (6), so as to reduce the resistance of the In_2_O_3_ sensor in the target gas. In our research, to confirm the excellent gas sensitivity of sensors, first, the chemical composition and electronic states of elements in 18-In_2_O_3_, 24-In_2_O_3_ and 48-In_2_O_3_ samples were studied by XPS. Then, the importance of adsorbed oxygen is verified by first-principles calculation. Finally, combined with the morphology and structure characteristics of the samples, the excellent methane sensing mechanism of the 48-In_2_O_3_ sensor we prepared was analyzed thoroughly and comprehensively.

O_2_(ads) + e^−^ → O_2_^−^ (ads), (T < ~100 ℃) (3)

O_2_^−^ (ads) + e^−^ → 2O^−^ (ads), (T < ~200 ℃) (4)

CH_4_ + 2O_2_^−^ (ads) → CO_2_(gas) + 2H_2_O(gas) + 2e^−^, (T < ~100 ℃) (5)

CH_4_ + 4O^−^ (ads) → CO_2_(gas) + 2H_2_O(gas) + 4e^−^, (T < ~200 ℃) (6)

**Figure 8:**
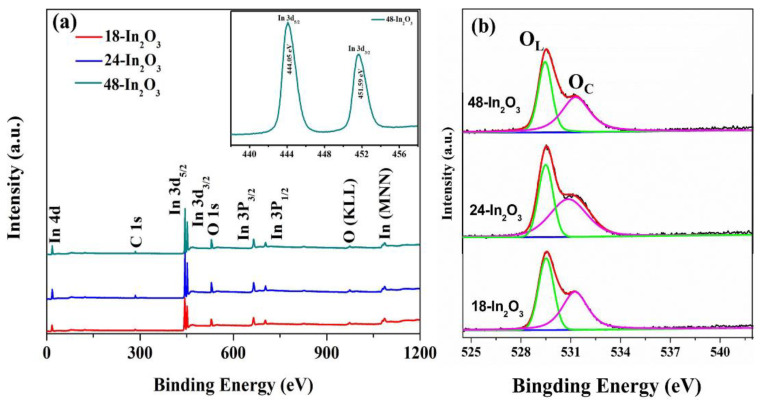
XPS spectrum of the porous In_2_O_3_ samples: (**a**) survey (the inset is an In 3d spectrum of the 48-In_2_O_3_) and (**b**) O 1 s.

As is shown in Figure 8a, In and O elements were detected in these three samples, and the C peak was the C contamination introduced during the XPS spectrum characterization [32]. The inset of Figure 8a displays the high resolution In 3d XPS spectrum of the 48-In_2_O_3_ sample. One can see that the In element is detected with two peaks at the binding energies of 444.05 eV and 451.59 eV, assigned to In 3d_5/2_ and In 3d_3/2_, respectively. The energy separation was 7.54 eV, which is consistent with In_2_O_3_, indicating that the oxidation state of In is In^3+^ [33]. Figure 8b shows the high-resolution O 1 s spectra of the three samples for further analysis. The O 1s spectra of the three samples can be fitted to three oxygen peaks, corresponding to lattice oxygen (O_L_) and adsorbed oxygen (O_C_). It has been reported that adsorbed oxygen plays a key role in the gas sensing reaction on the surface of semiconductor oxides by regulating the interaction with the target gas [31,32,34]. The adsorbed oxygen content of the three samples is shown in Table 3. With the prolongation of the hydrothermal reaction time, the percentage of adsorbed oxygen increased gradually. When the hydrothermal reaction reached 48 h, the highest content of adsorbed oxygen was obtained in 48-In_2_O_3_. The higher the content of adsorbed oxygen, the more reaction sites can be provided, thus improving the gas sensitivity.

The CASTEP package based on the first-principles calculation of density functional theory (DFT) was used to calculate the adsorption and activation capacity of CH_4_ molecules on the In_2_O_3_ (110) surface (with oxygen vacancies) in the presence or absence of adsorption oxygen (see the Supplementary Information and Figure S2 for computation details). The calculated results are shown in Table 4. Obviously, the adsorption energy of CH_4_ molecules on the In_2_O_3_ (110) surface is positive in the absence of adsorbed oxygen, which indicates that the material could not spontaneously adsorb CH_4_ molecules in this case. In the presence of adsorbed oxygen, the adsorption energy of the CH_4_ molecule on the In_2_O_3_ (110) surface is negative, and the absolute value of adsorption energy is relatively large. Moreover, the C-H bond length of the CH_4_ molecule is obviously prolonged after adsorption in the presence of adsorbed oxygen, which indicates that the material can not only spontaneously adsorb methane molecules, but also activate the CH_4_ molecule adequately. Therefore, the presence of adsorbed oxygen makes it easier for In_2_O_3_ nanomaterials to adsorb methane molecules and activate them more fully so as to improve the gas sensing performance of the sensor. This conclusion also proves that the sensitivity of CH_4_ is controlled by the theoretical model of adsorbed oxygen.

**Figure 9:**
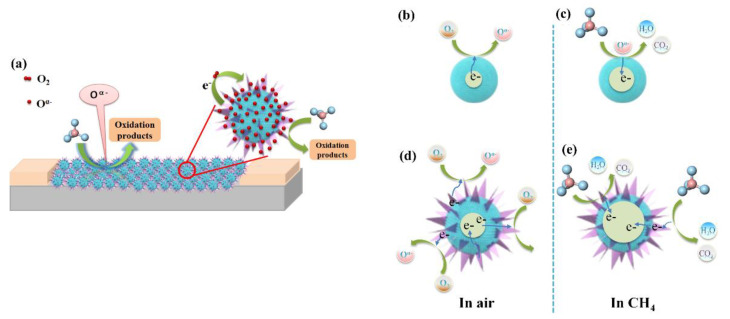
Schematic of the CH_4_ sensing mechanism (**a**) and changes in electronic structure for 48-In_2_O_3_ (**b**–**e**).

In this work, the excellent gas sensing performance of the In_2_O_3_ sensor is mainly controlled by the surface adsorbed oxygen model (the more intuitive sensitive mechanism behavior is shown in Figure 9). On the one hand, the 48-In_2_O_3_ sample has the highest adsorbed oxygen content, which will increase the number of active sites to promote more CH_4_ gas molecules to undergo redox reactions with it and release more electrons to return into the conduction band of the gas-sensitive material, so as to further increase the resistance change of the material and improve the gas sensing performance of the material. Moreover, from the above characterization analysis, it can be seen that 48-In_2_O_3_ has obvious characteristics of porous and hierarchical structure, which not only make the samples have a large specific surface area, but also, the pore structure is conducive to the diffusion and reaction of CH_4_ gas. Hierarchical structure can effectively reduce the possibility of particle agglomeration, improve the stability of the materials, and the three-dimensional hierarchical structure of 48-In_2_O_3_ can improve the regularity and directionality of carrier movement, CH_4_ gas molecules can easily enter the material and participate in the gas sensing reaction, and they can have a great impact on the gas sensing performance [12,13]. At the same time, we believe that the excellent sensitivity of the prepared 48-In_2_O_3_ sensor to CH_4_ is also controlled by the gas diffusion model. According to the pore size distribution test of the sample (as shown in Figure 4), the pore size of the sample is less than 10 nm. The diffusion behavior of gas molecules can be described by Knudsen diffusion theory [35,36].

## 4. Conclusions

In summary, the novel porous hierarchical In_2_O_3_ nanostructure that evolved with the extension of hydrothermal reaction time was successfully prepared by the hydrothermal and self-pore-forming method. The sensors based on the porous hierarchical In_2_O_3_ not only possess a lower optimal working temperature (40 °C), but also have a higher response value for methane detection, and the 48-In_2_O_3_ based sensor exhibits the best gas sensing performance. In addition to the above two advantages, the 48-In_2_O_3_ based sensor also has better repeatability, long-term stability, humidity resistance and response-concentration exponential linear relationship. The good gas sensing performance of a porous hierarchical 48-In_2_O_3_ sensor may be mainly due to its special nanostructure with both porous and hierarchical structure characteristics. The porous hierarchical nanostructure increases the number of active sites on the surface of the sensitive material, effectively improves the stability of the material and further improves the regularity and directionality of carrier motion, and all of these advantages make methane gas molecules easily diffuse and react and achieve high methane sensing performance. Although the sensor we fabricated can detect CH_4_ gas at near room temperature (30 °C), there are still some drawbacks to be addressed, such as long response–recovery time and insufficient selectivity. This will greatly affect the practical application of the sensor. In the follow-up research, we will take a series of methods including morphology control, construction of heterojunction and doping modification to improve the selectivity and response–recovery time.

## Figures and Tables

**Table 1:** Structural parameters of the porous In_2_O_3_ samples.

Materials	Hydrothermal Time (h)	D (nm)	Lattice Strain
18-In_2_O_3_	18	14.7	0.0029
24-In_2_O_3_	24	14.8	0.0023
48-In_2_O_3_	48	15.7	0.0022

**Table 2:** Comparison of CH_4_ sensing performance in this work with previous reported results.

Samples	Response (R_a_/R_g_)	Temperature (°C)	Concentration (ppm)	Specific Surface Area (m^2^·g^−1^)	Response/Recovery Time (s)
SnO_2_-Pd [18]	4.4	180	200	-	90/90
rGO-SnO_2_ [19]	1.6	200	1000	-	32/19
SnO_2_@rGO [20]	1.11	150	1000	110.55	61/330
ZnO–rGO [21]	1.047	190	4000	-	30/40
Ni_2_O_3_-SnO_2_ [22]	2.27	400	200	-	-
SnO_2_ QDs/CNHs [23]	2.5	90	1000	-	1074/1080
SnO_2_ nanorods [24]	1.05	100	500	-	13/-
RGO/ZnO [25]	1.67	250	500	-	-
SnO_2_ nanocrystals [26]	1.21	350	500	-	-
48-In_2_O_3_ (this work)	10.4	30	500	59.635	600/280

**Table 3:** Comparison of the adsorbed oxygen content and gas sensitivity of the In_2_O_3_ samples.

Materials	Adsorbed Oxygen	Operating Temperature (°C)	Response (R_g_/R_a_)
18-In_2_O_3_	43.1%	30	2.92
24-In_2_O_3_	46.7%	30	4.8
48-In_2_O_3_	50.5%	30	10.4

**Table 4:** Comparison of adsorption and activation abilities of methane molecules on the In_2_O_3_ (110) surface (with oxygen vacancies) in the presence or absence of adsorbed oxygen.

	Adsorption Energy (eV)	C-H Bond Length (Å)	
		Before Adsorption	After Adsorption
Absence of adsorption oxygen	6.751	1.097	1.179
Presence of adsorption oxygen	−3.079		3.394

## Data Availability

Not applicable.

## Supplementary Materials

The following supporting information can be downloaded at: https://www.mdpi.com/article/10.3390/nano12173081/s1, References [37,38,39,40,41,42] are cited in the Supplementary Materials. Figure S1: TG-DTA curves of the prepared In(OH)3-precursor; Figure S2: The calculated model of In_2_O_3_ (110), (blue ball represents Sn atom, yellow ball represents O atom).
