# Supplementary material for: Synthesis of Porous Hierarchical In2O3 Nanostructures with High Methane Sensing Property at Low Working Temperature
Source: Nanomaterials (Basel). 2022 Sep 5;12(17):3081. doi: 10.3390/nano12173081 (PMC9458147; doi:10.3390/nano12173081)
Supplement: Supplementary file 1 [file nanomaterials-12-03081-s001.zip › nanomaterials-1836488-supplementary.pdf]

nanomaterials

Supplementary Material

Synthesis of Porous Hierarchical In_2O_3 Nanostructures with High Methane Sensing Property at Low Working Temperature

Huiju Zhang [1], Jiangnan Chang [1], Yan Wang [2] and Jianliang Cao [1,*]

[1] College of Chemistry and Chemical Engineering, Henan Polytechnic University, Jiaozuo 454000, China
[2] State Collaborative Innovation Center of Coal Work Safety and Clean-Efficiency Utilization, Henan Polytechnic University, Jiaozuo 454000, China
* Correspondence: caojianliang@hpu.edu.cn

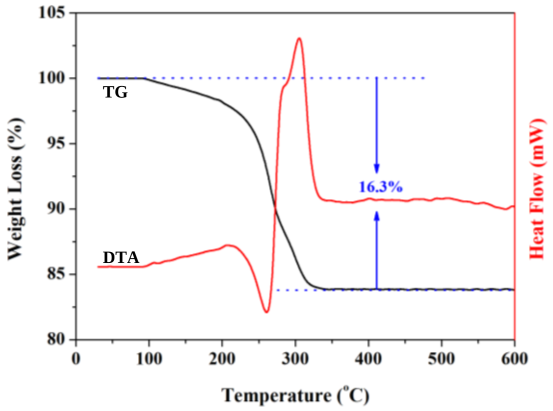

Figure S1. TG-DTA curves of the prepared In(OH)3-precursor.

In order to determine the calcination temperature for the synthesis of In_2O_3, the precursor $In(OH)_3$ synthesized by hydrothermal method was tested by thermal analysis. As shown in Figure S1, with the temperature increasing from 30 to 600 °C, a big weight loss (16.3 %) was observed in the TG curve, being consistence with the theoretical weight loss (16.28%) for the decomposition of $In(OH)_3$. Such a weight loss corresponds two continuous phase transformation processes, a endothermic process at around 265 °C and a exothermic process at around 305 °C. When the temperature is higher than 340 °C, the TG and DTA curves become of flat, indicating the formation of thermally stable In_2O_3. Based on above results, the calcination temperature of the precursor $In(OH)_3$ was set at 400 °C to ensure the formation of In_2O_3 with good crystallinity.

The DFT computations were carried out using Cambridge Sequential Total Energy Package (CASTEP) program package [1]. At the level of the Generalized Gradient Approximation (GGA), the exchange-collection functional of Perdew–Burke–Ernzerhof (PBE) was employed [2,3]. We used a 400 eV plane-wave cutoff and a 3 × 3 × 1 k-point Monkhorst-Pack mesh in the geometry optimization and energy calculations. The vacuum between slabs was set up to 15Å to preclude the interaction of the slabs and a slab model of ten-layer-thick In_2O_3 (110) was used. The super cell is expanded to 2 × 2, then the bottom six layers are fixed, and the top four layers are fully relaxed. The calculated model of In_2O_3 (110) is obtained as shown in Figure S2. Oxygen defects are bound to form on the surface of In_2O_3 after high temperature heat treatment during sample preparation [4]. Therefore, calculate the adsorption capacity of oxygen molecules and methane molecules on the surface of In_2O_3 (110) containing oxygen defects. It is calculated that the oxygen-containing defect In_2O_3 (110) surface has an adsorption energy of −2.492 for oxygen molecules, indicating that the In_2O_3 (110) surface can adsorb oxygen molecules spontaneously and has a strong adsorption capacity. The adsorption energy (ΔE_{ads}) of CH_4 molecule adsorbed on In_2O_3 (110) defect surface is calculated as follows:

$$\Delta E_{ads} = E_{ads} - E_{gas} - E_{def}$$

where E_{ads} represents the total energy of CH_4 molecule adsorbed on In_2O_3 (110) defect surface, E_{gas} and E_{def} represent the total energy of gas molecule (CH_4) and In_2O_3 (110) defect surface before adsorption, respectively. In general, the

negative value of ΔE_{ads} indicates that the gas molecule adsorption reaction is exothermic and can occur spontaneously under certain conditions, so the adsorption system is energy stable [5,6].

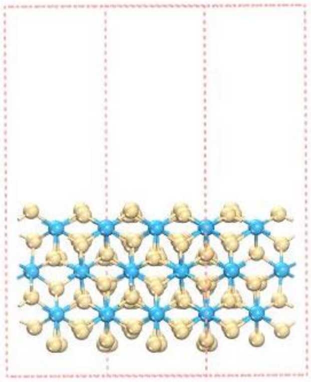

Figure S2. The calculated model of In_2O_3 (110), (Blue ball represents Sn atom, yellow ball represents O atom).

References

1. Payne, M.C.; Teter, M.P.; Allan, D.C.; Arias, T.; Joannopoulos, J. Iterative minimization techniques for ab initio total-energy calculations: molecular dynamics and conjugate gradients. *Rev. Mod. Phys.* **1992**, *64*, 1045–1097.
2. Perdew, J.P.; Chevary, J.; Vosko, S.; Jackson, K.A.; Pederson, M.R.; Singh, D.; Fiolhais, C. Atoms, molecules, solids, and surfaces: applications of the generalized gradient approximation for exchange and correlation. *Phys. Rev. B* **1992**, *46*, 6671–6687.
3. White, J.; Bird, D. Implementation of gradient-corrected exchange-correlation potentials in Car-Parrinello total-energy calculations. *Phys. Rev. B* **1994**, *50*, 4954–4957.
4. Park, S.; Ahn, H.-S.; Lee, C.-K.; Kim, H.; Jin, H.; Lee, H.-S.; Seo, S.; Yu, J.; Han, S. Interaction and ordering of vacancy defects in NiO. *Phys. Rev. B* **2008**, *77*, 134103.
5. Wu, G.X.; Zhang, J.; Wu, Y.; Li, Q.; Chou, K.; Bao, X. Adsorption and dissociation of hydrogen on MgO surface: a first-principles study. *J. Alloys Compd.* **2009**, *480*, 788–793.
6. Zeng, W.; Liu, T.M.; Liu, D.J.; Han, E.J. Hydrogen sensing and mechanism of M-doped SnO_2 (M = Cr^{3+}, Cu^{2+} and Pd^{2+}) nanocomposite. *Sens. Actuators B* **2011**, *160*, 455–462.
